# Bacterial Root Endophytes: Characterization of Their Competence and Plant Growth Promotion in Soybean (*Glycine max* (L.) Merr.) under Drought Stress

**DOI:** 10.3390/ijerph18030931

**Published:** 2021-01-21

**Authors:** Anamika Dubey, Diksha Saiyam, Ashwani Kumar, Abeer Hashem, Elsayed Fathi Abd_Allah, Mohammed Latif Khan

**Affiliations:** 1 Metagenomics and Secretomics Research Laboratory, Department of Botany, Dr. Harisingh Gour University (A Central University), Sagar 470003, MP, India; anamikadubey909@gmail.com (A.D.); khanml61@gmail.com (M.L.K.); 2 Department of Biotechnology, Dr. Harisingh Gour University (A Central University), Sagar 470003, MP, India; dikshusaiyam.96@gmail.com; 3 Botany and Microbiology Department, College of Science, King Saud University, P.O. Box 2460, Riyadh 11451, Saudi Arabia; habeer@ksu.edu.sa; 4 Mycology and Plant Disease Survey Department, Plant Pathology Research Institute, ARC, Giza 12511, Egypt; 5 Plant Production Department, College of Food and Agricultural Sciences, King Saud University, P.O. Box 2460, Riyadh 11451, Saudi Arabia; eabdallah@ksu.edu.sa

**Keywords:** drought stress, plant growth promotion, endophytic bacteria, biocontrol activity, agriculture productivity

## Abstract

Recently, the application of endophytes in the alleviation of different types of stresses has received considerable attention, but their role in drought stress alleviation and growth promotion in soybean is not well-stated. In this study, twenty bacterial endophytes were isolated from soybean root tissues and screened for plant growth-promoting (PGP) traits, biocontrol potential, and drought stress alleviation. Out of them, 80% showed PGP traits, and 20% showed antagonistic activity against *Fusarium oxysporum* (ITCC 2389), *Macrophomina phaseolina* (ITCC 1800), and *Alternaria alternata* (ITCC 3467), and only three of them showed drought tolerance up to 15% (−0.3 MPa). Results indicated that drought-tolerant PGP endophytic bacteria enhanced soybean seedling growth under drought stress conditions. Morphological, biochemical, and molecular characterization (16S rRNA) revealed that these three bacterial isolates, AKAD A1-1, AKAD A1-2, and AKAD A1-16, closely resemble *Bacillus cereus* (GenBank accession No. MN079048), *Pseudomonas otitidis* (MW301101), and *Pseudomonas* sp. (MN079074), respectively. We observed that the soybean seedlings were grown in well-watered and drought-stressed soil showed the adverse effect of drought stress on morphological (stem length, root length, plant fresh and dry weight) as well as on biochemical parameters (a decline of photosynthetic pigments, membrane damage, etc.). However, soybean seedlings inoculated with these endophytes have improved the biomass significantly (*p* ≤ 0.05) under normal as well as in drought stress conditions over control treatments by influencing several biochemical changes. Among these three endophytes, AKAD A1-16 performed better than AKAD A1-2 and AKAD A1-1, which was further validated by the ability to produce the enzyme 1-aminocyclopropane-1-carboxylate (ACC) deaminase in the following order: AKAD A1-16 > AKAD A1-2 > AKAD A1-1. Scanning electron microscopy images also showed a bacterial presence inside the roots of soybean seedlings. These findings supported the application of bacterial root endophytes as a potential tool to mitigate the effect of drought as well as of fungal diseases on the early seedling growth of soybean.

## 1. Introduction

**Figure 1:**
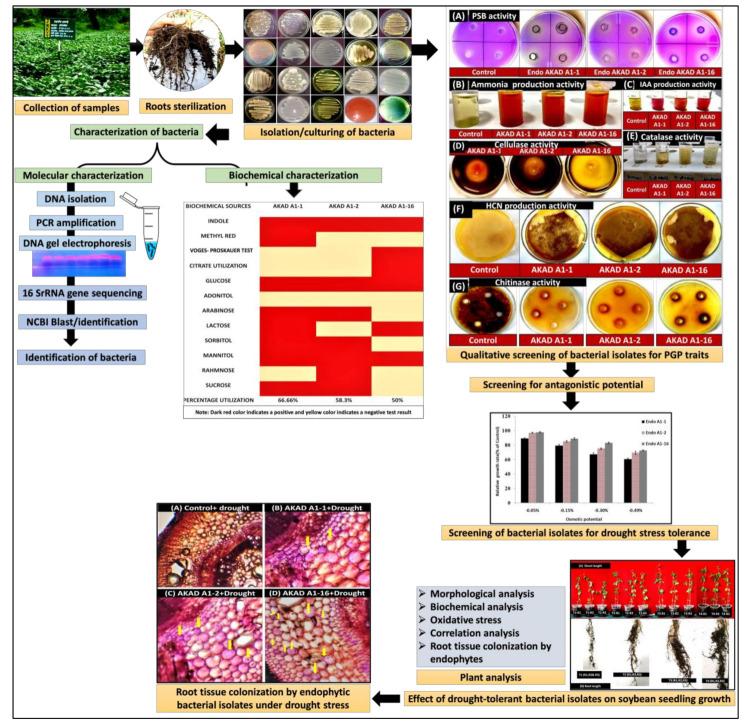
Flowchart of the experimental work [PSB, phosphate solubilizing bacteria; HCN, hydrogen cyanide].

Improving crop productivity in stressful conditions and sub-optimal sites will be a necessity to feed the rapidly growing human populations in the coming years. Increasing the production of nutrient-rich super-food crops to address and reduce several human health problems is of great interest to medical researchers and agriculturists [1]. Therefore, the promotion and use of a protein-rich leguminous crop like soybean (*Glycine max*) would be very helpful. Soybean is an economically essential crop cultivated in more than one hundred countries around the world [2]. The major producers are the United States (85,285,682.8 tons), Brazil (58,393,680.84 tons), Argentina (35,614,598.24 tons), China (14,408,902.84 tons), and India (8,878,848 tons), along with Canada, Bolivia, Paraguay, Ukraine, and others [3]. Environmental problems, such as global warming and climate change, have increased the levels of abiotic stresses. These indications also suggest that climate change will also expand the pathogens’ host range and increase the hazards of virulent strain development in future [4]. Plants in nature are constantly subjected to several abiotic stresses, including drought stress, that reduce annual crop yields up to 40% in some regions of the world [5]. Drought stress harms the plant’s water potential and cell turgor; this disrupts normal plant metabolism and alters the morphological and physiological traits of plants [6]. The growth and production of soybean plants are drastically hampered when exposed to drought stress conditions as soybean is a drought-sensitive crop [7]. Several physiological and morpho-biochemical parameters are adversely affected by water stress, including leaf wilting, leaf area reduction, root elongation, chlorophyll content, and the generation of reactive oxygen species (ROS) [8,9]. As a stress response, plants accumulate different osmoprotectants and osmolytes such as polyamines, betaine, proline, and sugars (trehalose, sorbitol, and mannitol) which are generally responsible for osmotic regulation under osmotic stress condition [10]. Apart from osmotic adjustment, these osmolytes have a dynamic role in retaining membrane integrity, mitigating ionic toxicity, protein stabilization, cellular component protection, scavenging ROS, and protecting antioxidant compounds as well as in balancing cellular redox under different abiotic stress conditions [11,12,13]. There are several changes that plants experience during water stress conditions, such as changes in relative water content, proline content, soluble sugars, proteins, lipid peroxidation, photosynthetic pigments, and antioxidant level [14,15]. The development of an eco-friendly strategy can be used to ameliorate the effect of drought stress in plants and there is an urgent need for such strategies in our agricultural systems, which have to cope with the impact of climate change [13,16]. Recently, several studies have advocated the role of endophytes, particularly of bacterial endophytes, in the alleviation of abiotic and biotic stress in crop plants by providing plants, several direct and indirect benefits [17,18]. Consequently, the incidence of combined abiotic and biotic stress is expected to be higher in the future [19]. The molecular mechanisms underlying abiotic and biotic stress tolerance have been intensely studied with much emphasis on the tolerance mechanisms concerning individual stresses [20]. However, in nature, plants are often simultaneously challenged by multiple biotic and abiotic stresses [21]. Therefore, the physiological and molecular responses that happen in plants exposed to a combination of concurrent abiotic and biotic stresses are still unexplored [22,23]. Endophytic bacteria receive considerable attention and may prove to be more important than rhizospheric bacteria in supporting plant health and growth because they are in more intimate contact with their host plants and can increase abiotic and biotic stress tolerance [24,25,26]. These bacteria can contribute in alleviating abiotic stresses of host plants via a variety of mechanisms like exopolysaccharide (EPS) production, dropping off the ethylene level utilizing 1-aminocyclopropane-1-carboxylate (ACC) deaminase which plays an important role in supporting plant growth under salinity and water stress conditions [27]. Other metabolites produced by PGP bacteria like phytohormones (auxins (or IAA), cytokinin), siderophores, volatile compounds, and organic acids that enhance plant growth under stress conditions [2]. Several studies on the beneficial role of endophytic microbes in enhancing drought stress tolerance in a variety of crop plants like wheat [27,28], licorice [29], *Helianthus tuberosus* [30], rice [31], pepper [32], and others [33] have been previously conducted, but to date, there have been no studies reported the ability of drought-tolerant plant growth-promoting antagonistic endophytic bacteria to alleviate the negative effects of drought stress in soybean plants. However, there is an urgent need for understanding the role of endophytes in simultaneous abiotic and biotic stress tolerance of plants, as not many studies have been undertaken in this direction. We, therefore, hypothesized that soybean root-associated bacterial endophytes play a dual role as a bio-control agent and as bio-fertilizers under drought stress conditions. To address this hypothesis, we screened the drought-tolerant plant growth-promoting antagonistic endophytic bacteria from soybean roots and used them to ameliorate the effect of drought stress in the early seedling growth of soybean. Different morphological–biochemical parameters were examined on the soybean grown under well-watered and drought stress conditions. The experimental work performed is shown in Figure 1.

## 2. Materials and Methods

### 2.1. Sampling and Isolation of Endophytic Bacteria

Root samples were collected from soybean (*Glycine max*) plants growing in a field located at 23°49′44″ N latitude and 78°42′46″ E longitude in Jawaharlal Nehru Krishi Vigyan Kendra, Bhopal Road, Sagar (M.P.), India. The root samples served as the source material for the isolation of endophytic bacteria. Collected root samples were washed several times under tap water to remove the surface soil after drying at room temperature; root samples were surface-sterilized with 70% ethanol for 3–5 min, then surface disinfection was performed using 0.1% HgCl_2_ for 2 min; the roots were finally washed 5–6 times with double distilled water for 2–5 min following the method of Cao et al. [34]. The surface-sterilized root samples were cut into small pieces macerated using a sterile pestle and mortar, then serially diluted in sterile distilled water and spread-plated in nutrient agar (NA) media. Endophytic bacteria were isolated using a standard protocol described by Hameed et al. [35]. The plates were incubated for 2 days at 28 °C. Morphologically different bacterial colonies were selected and purified on nutrient agar plates. The soil at the collection site had an electrical conductivity of 0.32 dS/m, a pH of 7.4, contained 230.4 (kg/ha) of nitrogen (N), 0.61 (kg/ha) of organic carbon (OC), 15.4 (kg/ha) phosphorus, 257 (kg/ha) of potassium (K), 28.2 ppm of sulfur (S), 1.53 ppm of boron (B), 1.19 ppm of zinc (Zn), 1.25 ppm of copper (Cu), 5.28 ppm of iron (Fe), and 7.12 ppm of (Mn) [36].

### 2.2. Screening Bacterial Endophytes for Polyethylene Glycol (PEG) Tolerance

The osmotic tolerance of the bacterial isolates was evaluated by recording their growth in the nutrient broth medium amended with different concentrations of PEG6000 (0–20%) and incubated the cultures at 28 °C for 24 h under continuous shaking (200 rpm) [37]. Bacterial growth was estimated by recording the optical density of the cultures at 600 nm using a spectrophotometer. The relative growth rates at different water potentials (−0.05, −0.15, −0.30, and −0.49) were determined and the direct correlation between growth inhibition and water potential was calculated.

### 2.3. Screening Bacterial Endophytes for PGP Traits

#### 2.3.1. Phosphate Solubilization

A qualitative assessment of the ability of the bacterial isolates to solubilize phosphates was made by observing the size of clear halo zones surrounding the bacterial isolates growing on Pikovskaya’s agar media containing 10 g of glucose, 5 g of Ca_3_ (PO_4_)_2_, 5 g of MgCl_2_, 0.2 g of KCl, 0.1 g of (NH_4_)_2_SO_4_, 0.25 g of MgSO_4_·7H_2_O, 15 g of agar, 0.025 g bromophenol blue per liter, pH 7.0 [38]. A quantitative assessment of tri-calcium orthophosphate solubilization in a liquid medium (Pikovskaya’s agar) was done using a protocol described by Fogg and Wilkinson [39]. 

#### 2.3.2. Indole-3-Acetic Acid Production

Auxin production was determined using a protocol initially described by Gordon and Weber [40] and modified by Loaces et al. [41]. Bacterial strains were grown in the DEV tryptophan broth (Himedia, Mumbai, India) and incubated at 28–30 °C in the dark for approximately three days. Bacteria hydrolyze the tryptophan into indole, which later on reacts with the Salkowski reagent to form color [42]. This mixture was incubated at 30 °C for 15 min in the dark. The phytohormone was quantified by recording absorbance at 530 nm by using a microplate reader.

#### 2.3.3. Ammonia Production

Bacterial endophytes were evaluated for their ability to produce ammonia water-containing NaCl and peptone using the protocol of Cappuccino and Sherman [43]. The development of a yellow to dark-brown color is positive for ammonia production. Absorbance was recorded at 450 nm in three replicates using a microplate reader. The amount of ammonia produced by the bacterial isolates was determined using a standard curve generated for ammonium sulphate.

#### 2.3.4. ACC Deaminase Activity

For determining the ACC deaminase activity, endophyte strains were grown in sterilized minimal Dworkin and Foster (DF) salt media (DF salt contains 6.0 g Na_2_HPO_4_, 4.0 g KH_2_PO_4_, 0.2 g MgSO_4_·7H_2_O, 2.0 g citric acid, 2.0 g glucose, and 2.0 g gluconic acid per liter with the following trace elements: 124.6 mg ZnSO_4_·7H_2_O, 78.22 mg CuSO_4_·5H_2_O, 11.19 mg MnSO_4_·H_2_O, 10 mg MoO_3_, 10 mg H_3_BO_3_, 1 mg FeSO_4_·7H_2_O; pH 7.2) amended with 3 mM ACC instead of (NH_4_)_2_SO_4_ as a sole nitrogen source [44,45]. The inoculated plates were incubated at 28 °C for 72 h; the colonies growing on the plates were taken and purified by sub-culturing the isolates. Therefore, the quantitative estimation of the ACC deaminase activity was done spectrophotometrically in terms of α-ketobutyrate production at 540 nm by comparing it with the standard curve of α-ketobutyrate, which ranged from 0.1 to 1.0 μmol [46]. The protein estimation was done as per the Bradford assay [47]. One unit of the ACC deaminase activity was expressed as the amount of α-ketobutyrate liberated in nmol per milligram of cellular protein per hour.

#### 2.3.5. HCN Production

Qualitative determination of hydrogen cyanide (HCN) production by the endophytic bacterial isolates was determined using the method of Lorck [48]. Bacterial isolates were streaked on a nutrient agar plate which was supplemented with 4.4 g/L of glycine. Therefore, the production of cyanide was detected by placing Whatman filter paper No. 1 soaked in 0.5% picric acid on the underside of the petri dish lids. The development of brown to red color after incubation for four days indicated HCN production.

#### 2.3.6. Enzyme Production Activity

Catalase activity was determined by adding 100 µL of 3% hydrogen peroxide to 24 h-old bacterial cultures in Eppendorf tubes [49]. The formation of bubbles is considered positive for catalase activity. Cellulase activity was determined by spot-inoculating 24 h-old bacterial cultures on CMC (carboxymethyl cellulose) agar plates and incubating them at 28 °C for 2 days. Plates were then flooded with an iodine solution. The appearance of a clear yellow zone around the bacterial colonies indicated cellulose hydrolysis while a brown zone surrounding the bacterial colonies indicated that cellulose had not been hydrolyzed [50]. Chitinase activity was measured by following the protocol of Babashpour et al. [51]; therefore, the development of clear/halo zones were observed around growing bacterial colonies indicating hydrolysis of chitin.

#### 2.3.7. Screening for Antagonistic Activity

To screen for the antifungal activity, the dual plate culture method was used on potato dextrose agar (PDA) against three phytopathogenic fungal isolates, *Fusarium oxysporum*, *Macrophomina phaseolina*, and *Alternaria alternata* [52]. A fungal disc (2 mm) was placed in the center and bacterial isolates were inoculated at four opposing corners on the PDA plates. The cultures were incubated at 28 °C for 4–5 days, after which the diameter of the fungal and bacterial colony was measured. Antifungal activity was expressed as % inhibition rate: (rc − r)⁄rc × 100%, where rc = radius of fungal growth (control plate without bacteria), r = the radius in the presence of the bacterial isolate. Microscopic observations of the interaction with *F. oxysporum* were performed by using a drop of mixed culture placed on a clean glass slide to which a drop of lactophenol blue was added.

### 2.4. Identification of Endophytic Bacteria

#### 2.4.1. Biochemical Characterization

A combination of 12 biochemical tests (HiAssorted^TM^ KB002, HiMedia, Mumbai, India) was used for biochemical characterization of these bacterial isolates by following the instruction provided by the manufacturer [53]. These tests are based on the principle of color change, change in pH, and utilization of the substrate by bacterial isolates. The bacterial isolates used for these tests were isolated and purified. Only pure cultures were used, and the kit was opened aseptically. The prepared inoculums in the amount of 50 µL were added to each well by using the surface inoculation method. The kit was also inoculated by stabbing each well with a loopful of inoculums and incubation at 35 °C for 18–24 h and color changes were observed and recorded.

#### 2.4.2. Molecular Characterization

Genomic DNA was extracted from the selected bacterial isolates using a Bacterial gDNA mini kit (Xcelris Genomics, Gujrat, India) according to the manufacturer’s instructions and the quality of the extracted DNA was analyzed using 0.8% agarose gel. The purity of the DNA was checked by measuring the 260/280 nm absorbance on a UV–visible spectrophotometer (Systronics, Gujrat, India). PCR amplification of the 16S rRNA gene was performed using the 27F forward (AGAGTTTGATCMTGGCTCAG) and 1492R reverse (TACGGTACCTTGTTACGACTT) universal primer set by using an automated thermal cycler (Bio-rad, Hercules, California, USA) with the following PCR conditions: initial denaturation at 94 °C for about 5 min followed by 30 cycles of amplification at 94 °C for 30 s, annealing at 52 °C for about 60 s, extension at 72 °C for 2 min, elongation at 72 °C for 10 min [54]. The nucleotide sequences were compared against nucleotide/gene bank databases using the NCBI BLASTn programs to identify the closest known taxa (https://blast.ncbi.nlm.nih.gov/Blast.cgi). The gene sequences obtained for the isolates were also submitted to GenBank and given the accession numbers MN079048, MW301101, and MN079074.

### 2.5. Bacterial Endophytes: Soybean Seedlings Grown under Drought Stress Conditions

Soybean seeds (variety JS 20-34) were obtained from Jawaharlal Nehru Krishi Vigyan Kendra, Bamhori Seed Farm, Bhopal Road, Sagar (M.P.), India. Soybean seeds were surface-sterilized with 25% sodium hypochlorite for 15 min and 70% ethanol for 5 min followed by 10 rinses with sterile distilled water. The soil used for the pot experiment was mixed with sand, coco peat, and soil (ratio of 1:1:2) and autoclaved 2–3 times at 15 psi for 15 min [55]. The seeds were sown at the depth of 3–5 cm in plastic pots (12 cm diameter × 15 cm height). Pots for the different treatments were placed in a randomized manner to provide equal exposure. For bacterial inoculation, pure cultures of the three selected endophytes were grown in the nutrient broth (HiMedia, Mumbai, India) for 48 h at 28 °C with continuous shaking (180 rpm). The bacterial cell pellet was collected by centrifugation at 10,000 rpm for 5 min followed by washing with sterilized distilled water. A total of 100 mL/pot of a bacterial cell suspension (10^6^ CFU mL^−1^) of each isolate were individually applied as a soil drench after seven days from the day of seeding [56,57]. The bacterial suspension was applied only once at the onset of the experiment. There were three pots per treatment and three seedlings per pot (two parallel experimental setups). Pots were placed in the following conditions: 32 ± 2 °C temperature and 40–50% average relative humidity. Control (uninoculated) plants and endophytic bacteria-inoculated plants were watered for 21 days. To induce drought stress, in one set of the experiment, water was withheld until seedlings got wilted, and in another set of the experiment, all the plants were watered. Well-watered soybean seedlings with and without bacteria were considered as positive control, while soybean seedlings with or without bacteria under the drought stress were considered the negative control. Eight treatments were administered with each treatment containing nine replicates. The eight treatments were as follows: T1—control (uninoculated), well-watered; T2—*Bacillus cereus* (AKAD A1-1), well-watered; T3—*Pseudomonas otitidis* (AKAD A1-2), well-watered; T4—*Pseudomonas* sp. (AKAD A1-16), well-watered; T5—control (uninoculated), with drought stress, T6—*Bacillus cereus* (AKAD A1-1), with drought stress; T7—*Pseudomonas otitidis* (AKAD A1-2), with drought stress; T8—*Pseudomonas* sp. (AKAD A1-16), with drought stress. Morphological parameters such as root and shoot length, fresh and dry weight of the soybean seedlings were recorded.

For biochemical analysis of the plants (well-watered and drought stress), leaf samples were collected at the seedling stage, frozen in liquid nitrogen, and kept at −20 °C for further analysis.

### 2.6. Analysis of Morphological, Physiological, and Biochemical Plant Parameters

Six replicates of each treatment were collected and growth parameters like shoot length (SL), root length (RL), plant fresh weight (PFW), plant dry weight (PDW) were recorded for each soybean seedling under well-watered and drought stress conditions. Plant dry weight was measured after drying the plant samples (shoot, root, and leaves) in a hot air oven at 70 °C until a constant weight was achieved [58]. To study the effect of endophyte inoculation on the stomatal development of the host plant, a common stomatal imprint technique was used [59]. The leaves were collected from the youngest trifoliate that had formed before the drought stress and after the imposed drought period. The imprints were collected from the abaxial surface of the leaves and numbers of stomata were counted per unit area of a leaf by using a standard compound microscope. Three fields of view were observed and the variables were counted for each sample [60]. LRWC (leaf relative water content) was calculated using the following formula, where FW is the fresh weight of the leaf, DW is the dry weight of the leaf after drying, and TW is the turgid weight of the leaf soaked in distilled water for 4–5 h [61,62].

$$\mathrm{RWC}\%=\frac{\mathrm{FW}-\mathrm{DW}}{\mathrm{TW}-\mathrm{FW}}\times 100$$

#### 2.6.1. Biochemical Analyses

A sample of fresh leaves was taken from each treatment for estimation of photosynthetic pigments like chlorophyll a, chlorophyll b, and total chlorophyll was measured using the standard protocol [63]. For estimation of total proline, 500 mg of leaf tissue was homogenized in 3% sulfosalicylic acid (*w/v*) followed by centrifugation for 10 min at 10,000 rpm. Then, the supernatant was mixed with glacial acetic acid and acidic ninhydrin and incubated in a water bath at 100 °C for about 1 h. Then, the reaction was terminated by keeping samples in an ice bath. Proline was extracted with toluene, and absorbance was recorded at 520 nm using the standard protocol [64]. The concentration of proline was determined by using the standard curve of proline and expressed as µmol proline g^−1^ FW. Total soluble sugar content was measured using an anthrone reagent [65].

#### 2.6.2. H_2_O_2_ Content and MDA (Malonaldehyde) or Lipid Peroxidation

H_2_O_2_ (hydrogen peroxide) content was determined by homogenizing 500 mg of the leaf sample in 0.1% trichloroacetic acid (TCA) (*w/v*) followed by centrifugation for 15 min at 12,000 rpm; then, 0.5 mL of the supernatant was added to 1 mL of potassium iodide (1 M) and 0.5 mL potassium phosphate buffer (10 mM, pH 7.0). The absorbance was recorded at 390 nm and the concentration of H_2_O_2_ was calculated from the standard curve [66]. The obtained values were expressed as nmol g FW. However, the MDA or lipid peroxidation in leaves was measured by estimating the formation of the thiobarbituric acid (TBA) reactive substance. MDA content, a measure of lipid peroxidation, was determined as described by Hodges et al. [67]. Absorbance was recorded at 532 nm and 600 nm and MDA was expressed as nM MDA formed using an extinction coefficient of 155 mM^−1^/cm.

#### 2.6.3. Scanning Electron Microscopy (SEM) of Roots Colonized by Endophytes

The roots of 21-day-old bacterial inoculated soybean seedlings were rinsed 4–5 times with a sterile HEPES buffer (0.1 M) and then cut into 3–4 mm pieces. The root pieces were dehydrated at 4 °C in a graded series of alcohol (30–70%) and then fixed in 4% glutaraldehyde for 3 h at 4 °C. Root sections were then critical point-dried, mounted on a metal stub, sputter-coated with gold/palladium, and imaged using an FEI NOVA SEM 450 scanning electron microscope (United States). 

### 2.7. Statistical Analysis

Data from well-watered and drought-stressed soybean seedlings were statistically analyzed using a one-way ANOVA followed by Duncan’s multiple range test and the Pearson correlation analysis using SPSS software version 21 (SPSS Inc./IBM Corp., Chicago, IL, USA). A *p* ≤ 0.05 was used to determine significant differences between the treatments (represented by different letters).

## 3. Results

### 3.1. Screening, Identification, and Characterization of Endophytic Bacterial Isolates

**Figure 2:**
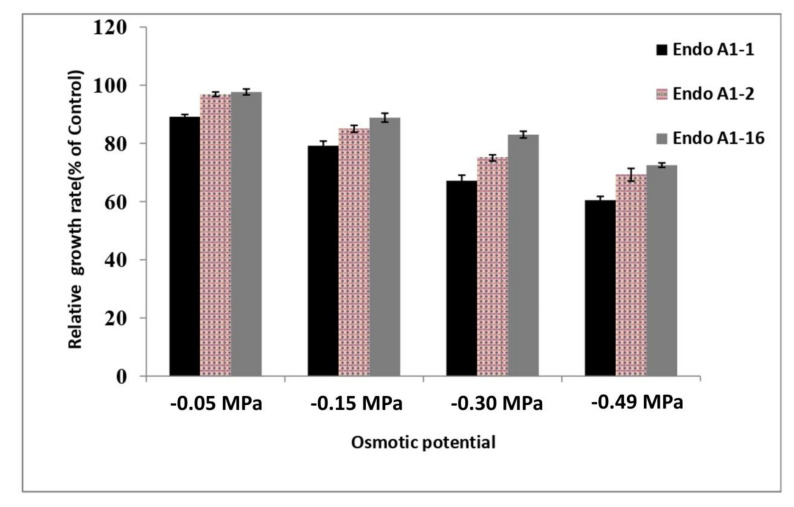
Effect of drought stress (−0.05 to −0.49 MPa) on the growth of bacterial isolates in the nutrient broth supplemented with 5–20% of PEG. Results expressed as % growth relative to the isolates grown in the nutrient broth not amended with PEG6000. The values are the means of three replicates ± standard deviation (*n* = 3).

Twenty bacterial endophytes were isolated from soybean root tissues and screened for various plant growth-promoting traits, bio-control potential, and drought stress tolerance (nutrient broth supplemented with 5–20% of PEG). Most of them showed PGP traits, but only three endophytic bacterial isolates (AKAD A1-1, AKAD A1-2, and AKAD A1-16) were observed to tolerate drought up to 15% (−0.3 MPa). These three were utilized for an in vivo pot experiment. The results indicated that endophytic bacterial strain AKAD A1-16 exhibited better stress tolerance at all levels of osmotic potential, which was presented as % growth relative to the control, followed by AKAD A1-2 and AKAD A1-1 (Figure 2). Optimum growth was observed by up to 15% PEG (−0.3 MPa).

**Figure 3:**
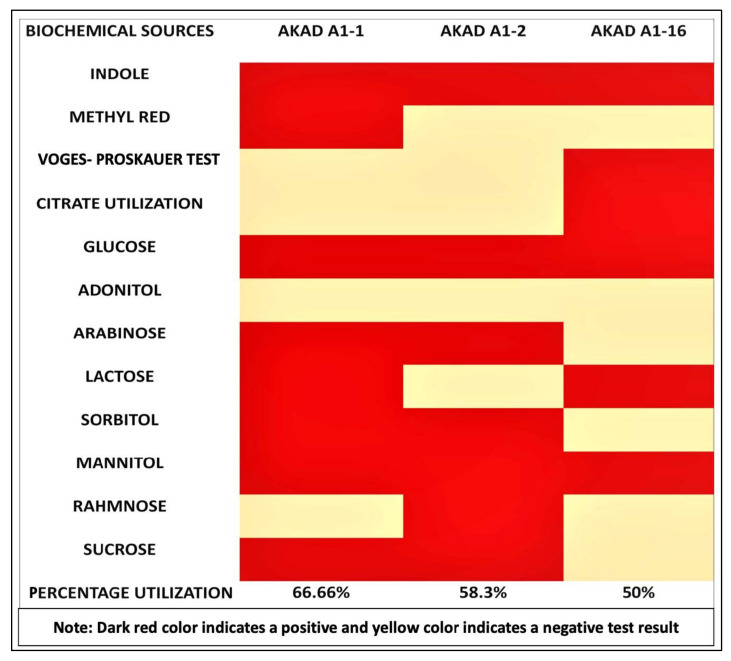
Biochemical source utilization by endophytic bacterial isolates.

Microscopic observations of these three bacterial isolates revealed that they were morphologically different. Biochemical characterization was performed on the utilization of 12 biochemical sources (utilization of indole, methyl red, voges-proskauer, citrate, adonitol, glucose, lactose, sorbitol, arabinose, mannitol, rhamnose, sucrose), which showed differences in their utilization patterns. Bacterial isolate No. AKAD A1-2 utilized 66.6% of the tested biochemical sources, AKAD A1-1 utilized 58%, and AKAD A1-16 utilized 50% (Figure 3). Based on 16S rRNA gene sequencing, endophytic bacterial strain AKAD A1-1 showed a close resemblance to *Bacillus cereus*; AKAD A1-2 and AKAD A1-16 were found to be affiliated with members of the genus *Pseudomonas* sp. The gene sequences were submitted to GenBank under the accession numbers MN079048, MW301101, and MN079074, respectively.

### 3.2. Characterization of Bacterial Endophytes for Plant Growth-Promoting Activity

**Figure 4:**
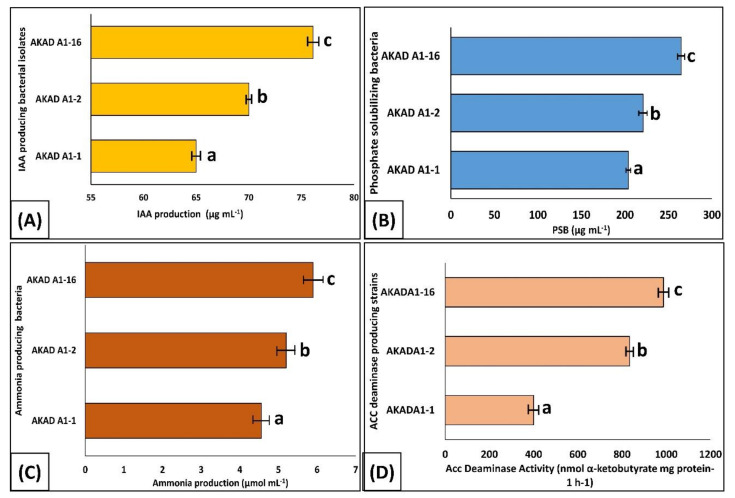
Plant growth-promoting activities of bacterial endophytes: (**A**) IAA production, (**B**) PSB activity, (**C**) ammonia production, (**D**) ACC deaminase activity. Note: Different letters in rows show that values are significantly different (*p* ≤ 0.05) from each other as evaluated using DMRT (Duncan’s multiple range test). The values are the means of three replicates ± SD (*n* = 3).

The results indicated that all three (AKAD A1-1, AKAD A1-2, and AKAD A1-16) bacterial endophytes produced more than 35 μg mL^−1^ IAA, with isolate AKAD A1-16 exhibiting the maximum level of IAA production at 71.2 μg.mL^−1^ (Figure 4A). All of the isolates also exhibited good results in the phosphate solubilization assay, solubilized more than 200 μg mL^−1^, with isolate AKAD A1-16 again exhibited the highest level of phosphate solubilization at 264.7 μg mL^−1^ (Figure 4B). Ammonia production by the isolates ranged between 4.5–6.0 μmol·mL^−1^. Isolate AKAD A1-16 produced the maximum amount of ammonia (5.9 μmol·mL^−1^), followed by AKAD A1-2 and AKAD A1-1 (Figure 4C). The highest ACC deaminase activity was recorded in the endophytic bacterial strain AKAD A1-16 (987.34 nmol α-ketobutyrate mg protein^−1^ h^−1^) followed by AKAD A1-2 (834.31 nmol α-ketobutyrate mg protein^−1^ h^−1^) and AKAD A1-1 (400.25 nmol α-ketobutyrate mg protein^−1^ h^−1^) (Figure 4D). All three isolates also exhibited positive results in production of chitinase, HCN, cellulase, and catalase activity (Figure 1).

### 3.3. Screening for Antagonistic Activity

**Figure 5:**
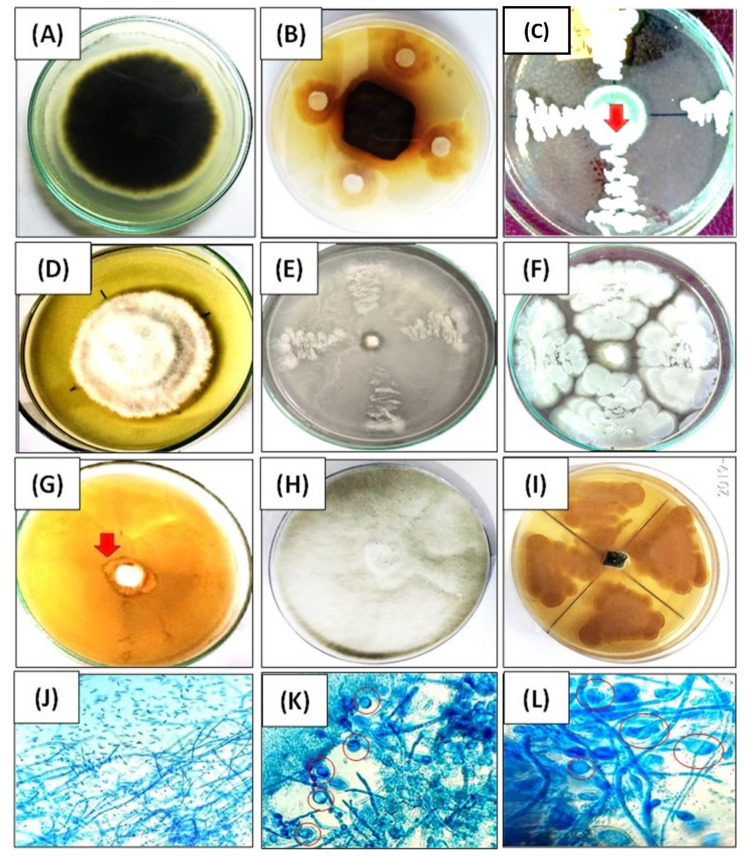
Antagonistic activity of the bacterial endophytes as determined in dual culture plates: (**A**) control plate *of A. alternata*; (**B**) AKAD A1-16 against *A. alternata*; (**C**) AKAD A1-1 against *A. alternata*; (**D**) control plate of *F. oxysporum*; (**E**) AKAD A1-1 against *F. oxysporum*; (**F**) bacterial strain AKAD A1-2 against *F. oxysporum*; (**G**) AKAD A1-16 against *F. oxysporum*; (**H**) control plate *of M. phaseolina*; (**I**) AKAD A1-16 against *M. phaseolina*; (**J**) microscopic observation of the control mycelium of *F. oxysporum*; (**K**) inhibition zone of AKAD A1-16 against *F. oxysporum* (40×); (**L**) inhibition zone of AKAD A1-16 against *F. oxysporum* (100×) (red circles represents chlamydospore formation).

The following bacterial endophytes, AKAD A1-1, AKAD A1-2, and AKAD A1-16, exhibited antagonistic activity against *F. oxysporum*, inhibiting fungal growth by 98.5%, 97%, and 98%, respectively. Isolates AKAD A1-1 and AKAD A1-16 also exhibited antagonistic activity against *A. alternata* by inhibiting fungal growth by 96% and 40.5%, respectively (Figure 5B–D). AKAD A1-16 showed > 90% inhibition against *M. phaseolina* (Figure 5H–J). The mycelia of *F. oxysporum* co-cultured with AKAD A1-16 exhibited abnormal mycelial morphology characterized by the development of a flocculated mycelium, hyphae with increased vacuolation, and the formation of chlamydospore-like structures as compared to the normal growth morphology (Figure 5K,L). These results indicate that AKAD A1-16 exhibits mycolytic activity against *F. oxysporum* (Table 1).

### 3.4. Effect of Drought-Tolerant Endophytes on Soybean Seedling Growth

#### 3.4.1. Morphological and Biochemical Parameters

AKAD A1-16-inoculated plants showed a significant increase in shoot length by 47.19% and 71.85%, followed by AKAD A1-2-inoculated seedlings, which showed an increase in shoot length by 34.7% and 51.9% in the well-watered and drought stress conditions, respectively. Similarly, AKAD A1-1-inoculated plants showed a 28.8% and 45.5% increase in shoot length under the well-watered and drought-stressed conditions, respectively. We observed that endophyte-inoculated plants showed a 27.9–75% increase in plant fresh weight under the well-watered condition and a 40–77% increase in plant dry weight as compared to (uninoculated) controls under the drought stress condition. Stomatal density increased by 45.31–68.7% under drought stress conditions due to treatment with endophytes (Table 2).

There was a 35.3–48.41% increase in the leaf relative water content in endophyte-inoculated soybean seedlings under drought stress conditions as compared to that of the uninoculated control. Soybean seedlings inoculated with AKAD A1-16 exhibited a 23.5–46.8% and 28.57–69.28% increase in the total chlorophyll content as compared to uninoculated control plants under well-watered and drought-stressed conditions, respectively (Table 3). Inoculated soybean seedlings showed a 14.87–42.56%, 13.8–38.3%, and 17.5–66.81% increase in sugar, protein, and proline content, respectively, under drought stress conditions as compared to control seedlings. Significant differences were observed in the MDA and H_2_O_2_ content in control soybean seedlings and endophyte-inoculated soybean seedlings under the well-watered and drought-stress conditions. The MDA and H_2_O_2_ content in the well-watered and drought-stressed soybean seedlings is presented in Table 3. The seedlings inoculated with AKAD A1-16 had the MDA and H_2_O_2_ content reduced by 33.7% and 28.8%, respectively, under the well-watered conditions and by 36.65% and 42.5%, respectively, under drought stress conditions as compared to control plants.

#### 3.4.2. Correlation between Morphological, Biochemical, and Oxidative Stress Parameters

Inoculation of soybean seedlings with bacterial endophytes had a positive effect on the seedlings subjected to drought stress conditions. This premise is supported by conducting a Pearson correlation analysis of the different morphological, biochemical, and oxidative stress parameters that were measured (Table 4). The results indicated that shoot length was positively correlated with root length (0.913), plant fresh weight (0.940), plant dry weight (0.930), relative water content (0.778), total chlorophyll content (0.90), total soluble sugar (0.423), proline (0.250), and protein (0.550) and negatively correlated with MDA (4.97) and H_2_O_2_ (8.72). The Pearson correlation coefficients (r) were < 0.01 and < 0.05 for all of the compared attributes (Table 4).

### 3.5. SEM Imaging of Root Endophytes

**Figure 6:**
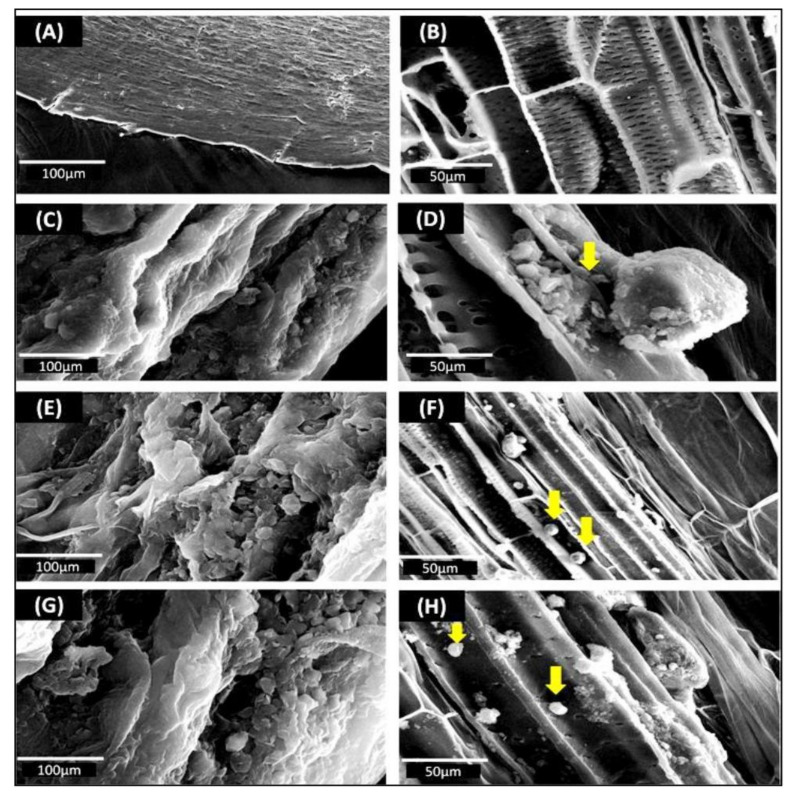
Scanning electron microscopic images of soybean seedling roots colonized by different endophytic bacteria under drought stress: (**A**) control uninoculated seedlings-at the root surface; (**B**) control uninoculated plant- root internal tissues; (**C**) AKAD A1-1- at the root surface; (**D**) AKAD A1-1—at the root internal tissue (yellow arrow); (**E**) AKAD A1-2—at the root surface; (**F**) AKAD A1-2—at the root internal tissue (yellow arrow); (**G**) AKAD A1-16—at the root surface (**H**) AKAD A1-16—at the root internal tissue (yellow arrow).

To validate the presence of bacterial endophytes inside the roots of soybean seedlings, SEM was performed, and images were analyzed (Figure 6A–H). No bacterial colonies were observed on the root surfaces (Figure 6A) or in the internal tissues (Figure 6B) of non-inoculated plants subjected to drought stress conditions. A population of 4.09 × 10^3^ CFU g^−1^, 2.6 × 10^4^ CFU g^−1^, and 3 × 10^3^ CFU g^−1^ was recorded for root samples of AKAD A1-1-, AKAD A1-2-, and AKAD A1-16-inoculated drought-stressed soybean seedlings, respectively. The seedlings inoculated with endophytic bacteria AKAD A1-1 and AKAD A1-16 showed biofilm formation inside the plant tissues (Figure 6D,H). The root colonization efficiency of bacterial endophytes was found to be in the order of AKAD A1-1 > AKAD A1-16 > AKAD A1-2.

## 4. Discussion

The result of this study supported the hypothesis that drought-tolerant bacterial endophytes display probiotic effects on soybean seedlings under drought stress conditions. These three bacterial endophytes improved soybean seedling growth significantly (*p* ≤ 0.05) under drought-stressed and normal soil conditions as compared to non-inoculated control plants. All the endophytes used in this study displayed multifarious PGP traits, including phosphate solubilization, auxin, ammonia, and ACC deaminase production, which may expedite plant growth directly, indirectly, or synergistically [68,69]. This may be due to the production of phytohormones by bacterial endophytes, which is considered the most acceptable mechanism in regulating plant growth and development [70]. Some bacterial strains can synthesize auxins (or IAA (indole-3-acetic acid)) in the root zone by using tryptophan as a secreted precursor molecule as root exudates are liable for root architecture [71]. Changes in root architecture induced by bacteria may lead to an intensification in the overall root surface area that subsequently enhances water and nutrient uptake, which may have beneficial effects on plant growth [58]. All the bacterial endophytes used in this study were tested as phosphate solubilizers, and our study was supported by the studies conducted by different researchers that demonstrated the role of phosphate solubilizing bacteria (PSB) in enhancing chlorophyll content and proline accumulation under drought stress conditions [72,73,74]. The production of ammonia through the conversion of molecular nitrogen by the nitrogenase enzyme has been shown to enhance plant growth by improving the plant roots’ architecture [56]. All endophytic bacterial strains used in the present study produced the ACC deaminase enzyme in the range of 400–1000 nmol α-ketobutyrate/mg protein^−1^/h^−1^. Drought stress might induce accelerated production of ethylene, which was reduced by inoculation of these bacterial strains having the ACC deaminase activity, which resulted in longer roots, which may be supportive in the uptake of more water from deep soil. Glick [75] reported ACC deaminase-producing bacteria to promote plant growth, as well as protect plants against salinity, drought, floods, flower wilting, organic pollutants, heavy metals, and also from both fungal and bacterial pathogens. These results are in accord with the findings of Danish et al. [76] who reported the application of ACC deaminase-producing *Pseudomonas aeruginosa* that can mitigate the adverse effect of drought in maize. Moreover, ACC deaminase-producing endophytic bacterial strains, *Bacillus cereus* strain AKAD A1-1, *Pseudomonas otitidis* strain AKAD A1-2, and *Pseudomonas* sp. strain AKAD A1-16 were able to grow in the presence of abiotic stresses generally allied with osmotic stress (20% PEG), suggesting that these bacteria can be active and hence express their PGP features in vivo under drought stress conditions. A study conducted by different scientists concluded that bacteria grow normally at an osmotic potential up to −0.3 MPa (15% of PEG) at higher concentrations of PEG; however, after that, bacterial growth gradually decreases [77,78,79]. Moreover, these drought-tolerant bacterial endophytes exhibited biocontrol activity against three soybean phytopathogens (*F. oxysporum*, *M. phaseolina*, and *A. alternata)* and provided evidence of the antagonism under drought stress. These bacterial isolates also show positive results for the production of cell wall-degrading enzymes such as cellulase, chitinase, and HCN that can hydrolyze fungal cell wall components consisting of proteins and chitin, β-1,3-glucanase; it has been shown to mediate positive response in the biocontrol of fungal pathogens [80]. Cellulase produced by beneficial bacteria plays a very important role in bacterial penetration inside the plant and helps in plant colonization [81]. The results of the present study provided encouraging results regarding the application of plant growth-promoting antagonistic endophytic bacteria with the drought-tolerant potential isolated from soybean roots.

Here in this study, we observed that inoculation with bacterial endophyte resulted in improved survival, plant biomass, and relative water content (*p* ≤ 0.05) as compared to those of normal soil conditions. These results corroborate the studies conducted on plant growth-promoting rhizobacteria (PGPR)-mediated amelioration of osmotic stress [77,78,79]. Loss of photosynthetic pigments has been reported to be linked with drought stress and chlorophyll concentration is recognized as an essential marker of drought stress tolerance in plants [58]. Endophyte-inoculated plants exhibited higher levels of leaf relative water content, photosynthetic pigments under the drought, and well-watered conditions compared to uninoculated plants (Table 3). In agreement with previous findings [5,17,58], we [64,65,82,83,84] observed a significant (*p* ≤ 0.05) increase in proline content in leaves of endophyte-inoculated soybean seedlings in response to drought stress. While proline accumulation is an extensive plant response towards environmental stresses, it is still debated if it is an indication of stress tolerance or stress damage [5]. Endophyte-inoculated soybean seedlings showed an increment in soluble sugar concentration under drought stress, suggesting that endophytic bacterial strains contribute to the accumulation of sugars for better osmotic adjustment by reducing drought-induced damage in the host plant. Notably, in this study, protein content was higher in endophyte-inoculated soybean seedlings than in uninoculated control seedlings; this may have been due to the formation of more defense-related proteins under drought stress conditions [72,85]. These results are in concordance with the study conducted by Gagné-Bourque et al. [5], wherein the authors reported *Bacillus subtilis* inoculation enhances plant biomass, RWC by the accumulation of proline, sugars, and amino acids in timothy plants under drought stress conditions. The linking between osmotic stress tolerance and proline accumulation has been extensively studied earlier by different scientists [64,65,82,83,84]. The primary response of the endophyte against reactive oxygen species is to enhance the accumulation of flavonoids, methionine, proline, sugars, proteins, and other phenolic compounds for enhancing plant tolerance. MDA is the final product of lipid peroxidation and its level can imitate the degree of cell membrane damage [27]. Soybean seedlings inoculated by bacterial endophytes had a significantly (*p* ≤ 0.05) decreased MDA and H_2_O_2_ content compared to those of the uninoculated control under drought stress conditions (Table 3). The increased level of oxidative stress may have resulted in a decrease in protein content caused by oxidative damage [86,87].

Efficient root colonization by inoculated bacteria is a critical step in the interaction between beneficial bacteria and host plants. Out of the three endophytic bacterial strains used in this study, AKAD A1-1 and AKAD A1-16 form a biofilm; this may be attributed to their intensive root colonization ability (Figure 6). Similar findings were also obtained in other studies where strains having good root/shoot colonization showed more promising results [28,71,77,88]. Colonization of roots by endophytic bacteria is a very important aspect of determining the survival of endophytic bacteria and the ability of the isolates to confer their PGP activity under stress conditions. Root colonization by PGPR strains was previously demonstrated using SEM [77,78,89]. Endophytic colonization may induce several physiological processes that help plants to sustain photosynthesis and growth under drought stress.

## 5. Conclusions

Studies on the role of fungal endophytes in plant growth promotion and bio-control of fungal pathogens have been performed extensively, but the dual potential of bacterial endophytes other than PGP traits is less explored. Thus, the overall study demonstrated that these endophytic bacterial strains can proficiently alleviate drought stress in soybean through various mechanisms: by improving plant growth, membrane integrity, water status, accumulation of compatible solutes, and osmolytes. This study utilized three PEG-induced drought stress-tested bacterial endophytes belonging to genera *Bacillus* and *Pseudomonas* for their potential for plant growth promotion under drought stress. To the best of our knowledge, this is the first report illustrating the role of drought-tolerant plant growth-promoting antagonistic endophytic bacteria in augmenting drought stress tolerance in the soybean plant. However, future investigations at the omics level are required to unravel the exact mechanism of their action to harness their potential in other crops for the fulfillment of the goal of sustainable crop production. The endophytes used in this study can likely be utilized for the development of bio-inoculants against drought stress conditions.

## Figures and Tables

**Table 1:** Antagonistic activity of the bacterial endophytes as determined by the dual culture plate assay.

Fungal Strain/Bacterial Strain	*Alternaria alternata*	*Fusarium oxysporum*	*Macrophomina phaseolina*
AKAD A1-1	96 ± 2.8%	98.5 ± 1.3%	No inhibition observed
AKAD A1-2	No inhibition observed	97 ± 2.27%	No inhibition observed
AKAD A1-16	40.5 ± 1.27%	98 ± 1.67%	90 ± 1.45%

The values are the means of three replicates ± SD (*n* = 3).

**Table 2:** Effect of inoculation of drought-tolerant bacterial endophytes on morphological parameters of soybean seedlings grown under well-watered and drought stress conditions.

Morphological Parameters					
Treatment	Shoot Length (cm)	Root Length (cm)	Plant Fresh Weight (g)	Plant Dry Weight (g)	Average Stomatal Density/Unit Area
T1	35.3 ± 2.04 ^b^	12.45 ± 0.8 ^b^	2.22 ± 0.10 ^b^	0.85 ± 0.03 ^b^	59.33 ± 2.34 ^b^
T2	45.10 ± 1.42 ^d^	15.74 ± 1.2 ^d^	2.74 ± 0.125 ^d^	1.27 ± 0.02 ^d^	80.5 ± 1.67 ^e^
T3	47.6 ± 1.01 ^e^	18.05 ± 1.5 ^f^	3.51 ± 0.2 ^g^	1.48 ± 0.01 ^f^	88.4 ± 2.3 ^f^
T4	52.03 ± 2.19 ^g^	22.03 ± 1.3 ^h^	3.89 ± 0.14 ^h^	1.60 ± 0.05 ^h^	90.5 ± 2.56 ^f^
T5	28.5 ± 1.50 ^a^	10.5 ± 0.78 ^a^	1.69 ± 0.03 ^a^	0.65 ± 0.03 ^a^	46.45 ± 1.45 ^a^
T6	41.49 ± 2.8 ^c^	13.83 ± 0.69 ^c^	2.4 ± 0.12 ^c^	1.09 ± 0.02 ^c^	67.5 ± 2.0 ^c^
T7	43.31 ± 1.9 ^cd^	17.16 ± 0.76 ^e^	2.9 ± 0.14 ^e^	1.35 ± 0.04 ^e^	71.5 ± 2.5 ^d^
T8	49.98 ± 1.5 ^f^	19.26 ± 1.2 ^fg^	3.05 ± 0.2 ^ef^	1.51 ± 0.02 ^g^	78.4 ± 1.4 ^e^

Note: the values are the means of six replicates ± SD (*n* = 6). The same letter within each column indicates no significant difference between the treatments (*p* ≤ 0.05) as determined by Duncan’s multiple range test. Treatments: T1—control (uninoculated), well-watered; T2—*Bacillus cereus* (AKAD A1-1), well-watered; T3—*Pseudomonas otitidis* (AKAD A1-2), well-watered; T4—*Pseudomonas* sp. (AKAD A1-16), well-watered; T5—control (uninoculated), with drought stress; T6—*Bacillus cereus* (AKAD A1-1), with drought stress; T7—*Pseudomonas otitidis* (AKAD A1-2), with drought stress; T8—*Pseudomonas* sp. (AKAD A1-16), with drought stress.

**Table 3:** Effect of inoculation of soybean seedlings with drought-tolerant bacterial endophytes on biochemical parameters under the well-watered and drought stress conditions.

Biochemical Parameters							
Treatment	LRWC %	Total Chlorophyll (mg g^−1^ FW)	Total Soluble Sugar (mg g^−1^ FW)	Proline (mg g^−1^ FW)	Protein (µg g^−1^ FW)	MDA (nmol g^−1^ FW)	H_2_O_2_ (mM g^−1^ FW)
T1	86.18 ± 1.8 ^bc^	1.73 ± 0.03 ^ab^	2.61 ± 0.04 ^a^	20.67 ± 1.8 ^a^	167 ± 3.13 ^a^	16.9 ± 1.7 ^d^	3.76 ± 0.05 ^c^
T2	90.26 ± 2.4 ^cde^	2.13 ± 0.02 ^cd^	2.79 ± 0.08 ^b^	24.7 ± 0.9 ^b^	204 ± 3.4 ^c^	14.6 ± 0.5 ^cd^	2.7 ± 0.08 ^b^
T3	91.81 ± 2.5 ^de^	2.4 ± 0.03 ^de^	2.84 ± 0.05 ^bc^	27.6 ± 0.62 ^bc^	209.6 ± 1.5 ^d^	13.2 ± 0.73 ^bc^	2.35 ± 0.05 ^a^
T4	93.86 ± 2.4 ^bcd^	2.54 ± 0.02 ^e^	3.24 ± 0.02 ^d^	30.52 ± 0.7 ^c^	218.3 ± 2.0 ^e^	11.2 ± 1.0 ^a^	2.16 ± 0.04 ^a^
T5	60.71 ± 3.33 ^a^	1.40 ± 0.04 ^a^	2.89 ± 0.01 ^c^	34.29 ± 1.4 ^d^	190.2 ± 2.1 ^b^	77.2 ± 2.30 ^h^	5.28 ± 0.25 ^d^
T6	83.5 ± 1.5 ^b^	1.80 ± 0.05 ^bc^	3.32 ± 0.09 ^d^	40.3 ± 1.5 ^e^	231.3 ± 1.5 ^f^	54.13 ± 1.8 ^g^	3.38 ± 0.07 ^c^
T7	87.18 ± 2.02 ^e^	1.85 ± 0.03 ^bc^	3.76 ± 0.06 ^e^	45.06 ± 1.6 ^f^	237.6 ± 1.5 ^g^	50.3 ± 1.57 ^f^	2.73 ± 0.14 ^b^
T8	90.1 ± 1.86 ^cde^	2.37 ± 0.04 ^de^	4.12 ± 0.12 ^f^	57.2 ± 1.9 ^g^	240.2 ± 1.7 ^h^	48.9 ± 1.70 ^e^	2.34 ± 0.05 ^a^

Note: the values are the means of six replicates ± SD (*n* = 6). The same letter within each column indicates no significant difference between the treatments (*p* ≤ 0.05) as determined by Duncan’s multiple range test. Treatments: T1—control (uninoculated), well-watered; T2—*Bacillus cereus* (AKAD A1-1), well-watered; T3—*Pseudomonas otitidis* (AKAD A1-2), well-watered; T4—*Pseudomonas* sp. (AKAD A1-16), well-watered; T5—control (uninoculated), with drought stress; T6—*Bacillus cereus* (AKAD A1-1), with drought stress; T7—*Pseudomonas otitidis* (AKAD A1-2), with drought stress; T8—*Pseudomonas* sp. (AKAD A1-16), with drought stress.

**Table 4:** Pearson correlation coefficients (r) between morphological, biochemical, and oxidative stress parameters of soybean seedlings, where SL—shoot length; RL—root length; PFW—plant fresh weight; PDW—plant dry weight; RWC—relative water content; CHL—chlorophyll; SUG—sugar; PRO—proline; PROT—protein; MDA—malonaldehyde; H_2_O_2_—hydrogen peroxide.

Correlation	Morphological Parameters				Biochemical Parameters					Oxidative Stress	
	SL	RL	PFW	PDW	RWC	CHL	SUG	PRO	PROT	MDA	H_2_O_2_
SL	1	0.913 **	0.940 **	0.930 **	0.778 **	0.902 **	0.423 *	0.250	0.550 **	−0.497 *	−0.872 **
RL		1	0.946 **	0.932 **	0.802 **	0.911 **	0.483 *	0.339	0.679 **	−0.440 *	−0.931 **
PFW			1	0.970 **	0.824 **	0.892 **	0.449 *	0.241	0.577 **	−0.545 **	−0.937 **
PDW				1	0.781 **	0.834 **	0.573 **	0.371	0.683 **	−0.411 *	−0.906 **
RWC					1	0.801 **	0.195	0.017	0.380	−0.709 **	−0.936 **
CHL						1	0.243	0.077	0.389	−0.655 **	−0.886 **
SUG							1	0.937 **	0.827 **	−0.419 *	−0.367
PRO								1	0.797 **	−0.607 **	−0.192
PROT									1	−0.295	−0.565 **
MDA										1	0.597 **
H_2_O_2_											1

** Correlation is significant at the 0.01 level (two-tailed). * Correlation is significant at the 0.05 level (two-tailed).

## Data Availability

The data presented in this study may be available on request from the corresponding author.
